# The COVID-19 epidemic, its mortality, and the role of non-pharmaceutical interventions

**DOI:** 10.1177/2048872620924922

**Published:** 2020-04-01

**Authors:** Niel Hens, Pascal Vranck, Geert Molenberghs

**Affiliations:** Data Science Institute, I-BioStat, Universiteit Hasselt, Belgium; Centre for Health Economics Research and Modelling of Infectious Diseases (CHERMID), Vaccine & Infectious Disease Institute (VAXINFECTIO), University of Antwerp, Belgium; Heart Centre Hasselt, Jessaziekenhuis, Belgium; Faculty of Medicine and Life Sciences, Universiteit Hasselt, Belgium; I-BioStat, KU Leuven, Belgium

**Keywords:** COVID-19, non-pharmaceutical, interventions, epidemiological modelling

## Abstract

COVID-19 has developed into a pandemic, hitting hard on our communities. As the pandemic continues to bring health and economic hardship, keeping mortality as low as possible will be the highest priority for individuals; hence governments must put in place measures to ameliorate the inevitable economic downturn. The course of an epidemic may be defined by a series of key factors. In the early stages of a new infectious disease outbreak, it is crucial to understand the transmission dynamics of the infection. The basic reproduction number (*R*_0_), which defines the mean number of secondary cases generated by one primary case when the population is largely susceptible to infection (‘totally naïve’), determines the overall number of people who are likely to be infected, or, more precisely, the area under the epidemic curve. Estimation of changes in transmission over time can provide insights into the epidemiological situation and identify whether outbreak control measures are having a measurable effect. For *R*_0_ > 1, the number infected tends to increase, and for *R*_0_ < 1, transmission dies out. Non-pharmaceutical strategies to handle the epidemic are sketched and based on current knowledge, the current situation is sketched and scenarios for the near future discussed.

## Introduction

The world has not seen an epidemic that turned into a pandemic without adequate medicinal products since the H1N1 pandemic in 1918 (Spanish flu).^1,2^ There are important similarities as well as key differences. Importantly, COVID-19 is *not* influenza, it is worse. COVID-19 has a wide spectrum of clinical severity, ranging from asymptomatic to critically ill, and ultimately death.^3–6^ A common and prominent complication of advanced COVID-19 is acute hypoxaemic respiratory insufficiency or failure requiring oxygen and ventilation therapies.^7,8^

**Figure 1.**
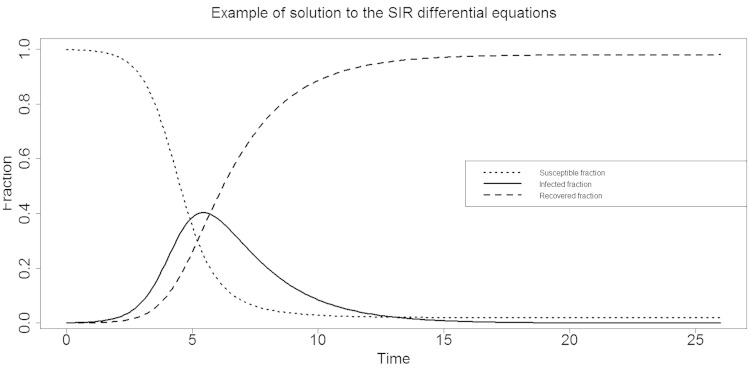
SIR model. Example of evolution of susceptible (S), infected (I) and recovered (R) fractions over time

A key difference between COVID-19 and seasonal influenza is the very different reproduction number, *b*,^9–11^ a key quantity that, together with the recovery rate, *k*, drives the evolution over time of the susceptible, infected and recovered fractions, S(t), I(t) and R(t), respectively. A graphical depiction of the simple so-called SIR model is given in Figure 1. If *b* <1.0, the epidemic dies out quickly. If *b* >1.0, the infected fraction evolves towards a peak before decreasing again. As can be seen from Figure 1, the initial evolution of the infected fraction is roughly exponentially shaped, prior to reaching the peak. Current-day modelling may involve additional compartments (e.g. susceptible – exposed – infected – recovered – susceptible) and factor in as much information as possible from other sources, such as contact information, data from serological surveys, etc.^10–12^

The reproduction number is very different between seasonal influenza, where it is usually around 1.5, and COVID-19, where it is estimated at about 2.5 if medication nor vaccines are available, and no non-pharmaceutical interventions are implemented.^1,13–15^ This was the number estimated, for example, in the early phases of the Hubei epidemic.^16^ A few other examples are as follows: for measles, the reproduction number is about 12–18, for mumps it is roughly 5 and for SARS around 2.5.^16–20^

An important task for the epidemiologist is to estimate *b*, especially in a newly emerging viral epidemic such as caused by SARS-CoV-2. Epidemiologists use the concept of *infectious period*, which in itself needs to be estimated from accruing data; they also use the *contact rate*, and finally the *mode of transmission*. For COVID-19, the dominant mode of transmission was established quickly as airborne droplets, while other routes such as faeces are possible.^9,10^ For the infectious period, reliable data need to be available. Also, it is not a constant, but depends on various factors, such as age, for example. Values of five days for the latency period and five days for the infectious period have been put forward,^1^ as well as four days for the serial interval,^21^ shorter than the incubation period and hence suggesting substantial pre-symptomatic transmission. We will turn to the remaining quantity, the contact rate, soon.

A key aspect is that the ‘recovered’ fraction also includes deaths. This requires careful attention from a public health standpoint. A death rate of, say, 0.5–1.0% translates in a population of 10 million people to 50,000–100,000 deaths. It is not just the case fatality rate (or the infection fatality rate) that causes distress and disruption, but evidently also the numbers needing intensive care or mechanical ventilation at a given point in time – the critically ill category.

The contact rate is the quantity we can and should have an impact on, especially in the absence of vaccines and treatment.^22–25^ There are three possible strategies. The first one is *suppression*. It essentially means that the reproduction number is forced below 1.0 by imposing very severe contact restrictions on the population, as was done in China minus Hubei. This is the quickest way to put out the fire. Of course, a large fraction of the population is then kept in the susceptible state, and measures should be in place to avoid the epidemic from flaring up, while monitoring very effectively so that, if it does, suppression measures can be enacted again. Clearly, China is in this situation, and likely will be until vaccines and medication are available.^11^ Cheap, widespread, sensitive and specific diagnostic tools help maintain control. Their quick development is also crucial.^11^

The second strategy is *mitigation*. Here, measures are taken to bring the reproduction number down to a level at which the epidemic is slowed sufficiently so that the number of critically ill cases at any time, *t*, can be handled by the health care system. It can be supplemented by a temporary capacity increase of the system (e.g. field hospitals, annexes to existing hospitals). The measures taken in Belgium aim to lower the reproduction number so that the health system can appropriately deal with COVID-19 patients. It is not merely numbers but also the severity of cases, even when non-fatal. Because of an epidemic’s initial exponential growth, even when it is off to a slow start, it is unfortunately true that small causes, such as lockdown parties, can have severe consequences. In addition, the measures will have the required effect if the population is truly closed, or part of a larger population with exactly the same population dynamics. Boundary effects, such as transnational contacts (e.g. between Norway and Sweden) can fatally undermine the mitigation strategy. Further, the earlier that contact rates are drastically reduced (severe social distancing), the better. The closer we come to cutting off the virus’s transmission mode, the sooner we will change, and hence flatten, the curve.

The third strategy, or absence thereof, is counting solely on *herd immunity* (*group immunity*).^25^ At face value, this appears to be a sensible strategy. It will typically produce a shorter epidemic than with mitigation, and afterwards the population will be immune at group level. That is, the fraction of recovered people (and hence immune for a certain time, e.g. the rest of the season) will be so large that a re-emerging virus will not find enough susceptible population members to push the reproduction number above 1.0, and the epidemic will soon extinguish. However, the area under the curve will increase, leading to considerable increase in critically ill patients and deaths.

**Figure 2.**
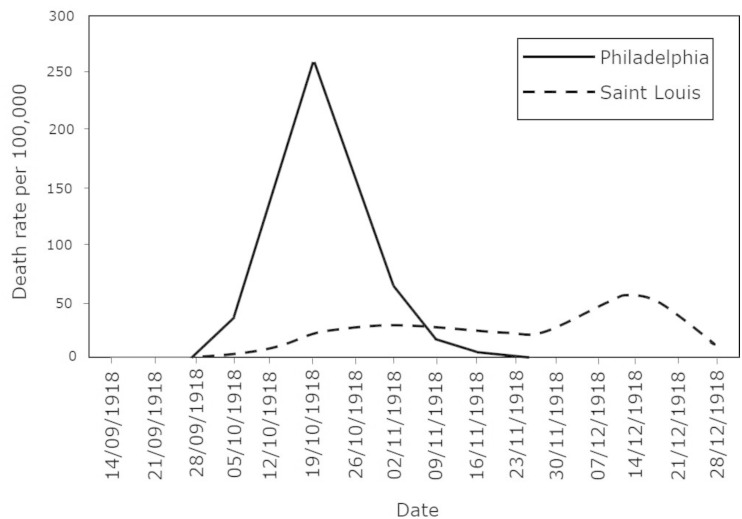
Death rate for every 100,000 people in Philadelphia and St. Louis between 14 September 1918 and 28 December 1918

Figure 2 shows the effect of reducing the contact rate, or not. Philadelphia ignored the warnings of an influenza epidemic among soldiers and organized a WWI-related parade. They closed the city a few days later, when all hospitals were filled to capacity. Mass events were evidently also prevalent in the early stages of the COVID-19 pandemic. St. Louis implemented what we now term *social distancing* immediately after detecting the first two cases. The number of deaths per capita was double in Philadelphia relative to St. Louis. Additionally, Philadelphia’s health care system was completely overwhelmed, while St. Louis was able to cope with the epidemic, which killed about 50 million people worldwide.

**Figure 3.**
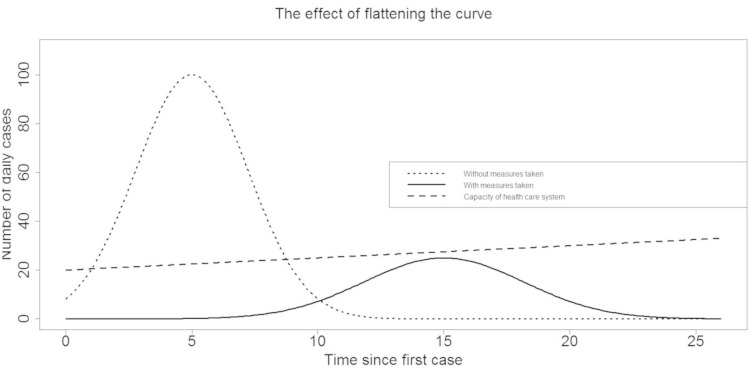
The effect of flattening the curve

Figure 3 depicts what happens if we move from a Philadelphia to a St. Louis scenario. The total volume of the epidemic will reduce, as the total fraction of infected population members is roughly equal to 1 – 1/*b*, but a much more important effect is that the number of infected cases at any point in time remains below the (perhaps enhanced) capacity of the health care system.

Recall that the number of cases is not relevant when considered in isolation. Much more important is the number of critical cases, and the fatality rate. Two very important remarks apply. First, the number of actual cases is very different from the number of confirmed cases. China implemented rigorous measures, as did South Korea, to identify cases. In Europe, this has been difficult to varying degrees during the epidemic onset and peak period. Undercount ratios are very different from country to country, implying that epidemiologists need to estimate the actual number of cases from the number of confirmed cases. There are ways to do so, but it adds further uncertainty to the predictions made. Second, the infection fatality rate will increase if the health care system is overwhelmed, as well as by the extent to which it does.

## Scenarios for some European countries

Estimation of the infection fatality rate is difficult because of the large group of asymptomatic and undiagnosed cases. For the case fatality rate, figures around 5% have been quoted, although some authors suggest much higher rates if longer time delays were to be taken into account.^25^ The infection fatality rate has been estimated to range over 0.3–1.0%.^23^ The immune fractions for Austria and The Netherlands have been estimated to be around 1% and 3%, respectively. Estimates based on samples from blood donors, for example, might slightly underestimate the quantity. For Belgium, 5000–6000 deaths against the background of 3–5% immunity would suggest an infection fatality rate (IFR) of 1–2%. Likely, the death rate is overestimated due to a very inclusive definition of COVID-19 related deaths. Should the original reproduction number of 2.5 be maintained, in an unmitigated scenario, and assuming an IFR of 1%, then roughly 60% would be infected, leading to 65,000 deaths. For a reproduction number of 1.5, roughly one-third of the population would become infected, leading to 35,000 deaths. Mid-April 2020, estimates of the reproduction number in various European countries indicate that it dropped below 1.0, due to social distance measures.

## Conclusions and outlook

The larger the immune fraction, the easier to contain the epidemic in the future. But this comes at the cost of a severely overwhelmed health care system.^26^ This can be avoided, and apparently has been, by drastic social distance measures. What will happen next? For this, it is important to recall a few key differences from influenza. Anderson et al.^25^ compare both on four aspects. First, the infection fatality rate is different and likely higher (about 0.1% for influenza). Second, there is infectiousness before the onset of symptoms. Current partial knowledge suggests a period of 1–2 days before onset, roughly like influenza. Third, with COVID-19, there may be up to 40–50% of mild or asymptomatic cases. Fourth, while influenza has a short infectious period of a couple of days, for COVID-19, although still relatively uncertain, it might be around 10 days.

Anderson et al.^25^ conclude that this produces a slowly emerging epidemic, which then accelerates, only to last longer than an influenza epidemic.^27^ Using mathematical modelling,^28^ Kissler et al.^1^ examine scenarios for the time period ahead, based on current knowledge, as well as realistic but as yet unverified scenarios based on knowledge from beta coronaviruses OC43 and HKU1, including the immune period, whether or not re-infection can take place, seasonality, cross-immunization with these other coronaviruses, and the length and severity of lockdown measures. In the absence of pharmaceutical interventions, depending on the scenario, annual, biennial, or even five-yearly outbreaks are expected. Such model-based predictions, even when there is considerable uncertainty, can support policy makers in developing a resilience strategy for the period until sufficiently adequate pharmaceutical interventions are possible. These may involve several time-related social distancing measures, preparedness to re-enter lockdown for certain periods, establishing quarantine procedures for individuals and groups, controlling contact between populations, within and between countries, et cetera. The measures taken are intimately linked to strategies aimed at building up some herd immunity in a controlled fashion.
